# Gut microbiota-derived gamma-aminobutyric acid improves host appetite by inhibiting satiety hormone secretion

**DOI:** 10.1128/msystems.01015-24

**Published:** 2024-09-24

**Authors:** Shouren Li, Mengqi Liu, Yao Han, Cong Liu, Shixi Cao, Yalei Cui, Xiaoyan Zhu, Zhichang Wang, Boshuai Liu, Yinghua Shi

**Affiliations:** 1 College of Animal Science and Technology, Henan Agricultural University, Zhengzhou, China; 2 Henan Key Laboratory of Innovation and Utilization of Grassland Resources, Zhengzhou, China; 3 Henan Forage Engineering Technology Research Center, Zhengzhou, Henan, China; University of California San Diego, La Jolla, California, USA

**Keywords:** feeding regulation, microbiota-gut-brain axis, gamma-aminobutyric acid, appetite hormones

## Abstract

Globally, appetite disorders have become an increasingly prominent public health issue. While short-term appetite loss may seem relatively harmless, prolonged instances can lead to serious physical and mental damage. In recent years, numerous studies have highlighted the significant role of the “microbiota-gut-brain” axis in the regulation of feeding behavior in organisms, suggesting that targeting the gut microbiota may be a novel therapeutic strategy for appetite disorders. However, the molecular mechanisms through which the gut microbiota mediates the increase in host appetite and the causal relationship between the two remain unclear. Based on this, we conducted 16S rRNA sequencing to analyze the gut microbiota of rabbits with high and low feed intake, followed by fecal microbiota transplantation (FMT) and metabolite gavage experiments to elucidate the underlying mechanisms. Our research indicates that the high feed intake group exhibited significant enrichment of the *g__Bacteroides* and gamma-aminobutyric acid (GABA), and intragastric administration of GABA effectively promoted the host’s feeding behavior. The underlying mechanism involves GABA derived from the gut microbiota inhibiting the secretion of satiety hormones to enhance the host’s feeding behavior. Furthermore, the results of FMT suggest that differences in gut microbiota composition may be a contributing factor to varying levels of feed intake in the host. In conclusion, these findings emphasize the role of the gut microbiota-derived GABA, in increasing host feed intake, offering a new target for the treatment of appetite disorders from the perspective of gut microbiota.

**IMPORTANCE:**

The incidence of anorexia is rapidly increasing and has become a global burden. Gut microbiota can participate in the regulation of host feeding behavior, yet the molecular mechanisms through which the gut microbiota mediates the increase in host appetite and the causal relationship between them remain unclear. In this study, we utilized 16S rRNA sequencing to investigate the composition of the gut microbiota in rabbits with varying levels of feed intake and employed fecal microbiota transplantation and gastric infusion experiments with gamma-aminobutyric acid (GABA) to elucidate the potential mechanisms involved. GABA derived from the gut microbiota can effectively enhance the host’s feeding behavior by inhibiting the secretion of satiety hormones. This discovery underscores the pivotal role of the gut microbiota in modulating host appetite, offering novel research avenues and therapeutic targets for appetite disorders.

## INTRODUCTION

Satiety and hunger, as the primary involuntary motives for feeding behavior in organisms, are controlled by the central nervous system (1–3). Among them, the hypothalamus, as one of the core nodes in regulating feeding, can integrate appetite signals from humoral and neural pathways, thereby promoting or inhibiting the organism’s feeding behavior (4). Food intake is a crucial component of energy metabolism, and normal feeding behavior is vital for maintaining metabolic homeostasis and overall organismal health. Prolonged appetite loss can lead to metabolic imbalance due to malnutrition, resulting in serious complications such as anemia, decreased immune function, and sleep disturbances (5, 6). Furthermore, compared with other acute and severe illnesses, the symptoms of appetite loss may not appear severe in the short term, leading modern individuals to often underestimate or even ignore the harm it can cause. Due to the complex pathogenesis of this condition and the relatively late start of relevant clinical and experimental research, ideal treatment outcomes have not yet been achieved.

In recent years, with the development of high-throughput sequencing methods, researchers have discovered that the gut microbiota can have a profound impact on the host’s energy metabolism and the occurrence and development of related diseases (7–9). First, significant changes in the composition of the gut microbiota have been observed in patients with obesity and anorexia nervosa compared with healthy control groups (10–12) and the results of further fecal microbiota transplantation (FMT) trials indicate a close relationship between the gut microbiota and the host’s feeding behavior (13). The application of anorexia mouse models and limited lysine pig models has provided evidence supporting the association between the gut microbiota and changes in hypothalamic appetite peptides and circulating satiety hormones (14, 15). Additionally, metabolites derived from the gut microbiota, such as short-chain fatty acids, bile acids, and indole derivatives, have long been believed by researchers to play a key role in the “microbiota-gut-brain” axis, which can affect physiological functions of the organism through a variety of mechanisms (16, 17). Therefore, reshaping the gut microbiota may be a promising strategy for treating appetite loss and other eating disorders. However, research on how the gut microbiota and its metabolites regulate the host’s feeding behavior is limited, and the causal relationship between the gut microbiota and host appetite remains unclear.

Therefore, in this study, we employed 16S rRNA gut microbiota sequencing on rabbits with varying feed intake levels to identify bacteria that may influence host appetite. Subsequently, we conducted FMT experiments to further elucidate the causal relationship between gut microbiota and host feeding behavior. The results indicated that the gut microbiota compositions differed between rabbits with high and low feed intake levels, suggesting that these differences largely contribute to the variations in host feeding levels. Additionally, we observed an enrichment of bacteria producing gamma-aminobutyric acid (GABA) in the high feed intake group and confirmed the inhibitory effect of GABA on gut satiety hormone secretion through gastric infusion experiments. These findings underscore the role of gut microbiota in modulating host feeding behavior and offer new strategies for future livestock production practices and the treatment of eating disorders.

## MATERIALS AND METHODS

### Animals and experimental design

#### Experiment 1: grouping based on high and low feed intake and experimental design

A total of 100 male New Zealand rabbits (30 days old, weighing 683.05 ± 16.35 g) were provided by Wuhan Wanqian Jiaxing Biotechnology Co. Ltd. (Wuhan, China). All rabbits were individually housed in cages with dry and ventilated conditions, with *ad libitum* access to feeds and water. Throughout the experiment, each rabbit was provided with a basic daily diet, the composition and nutritional levels of which can be found in Table S1. After a 7-day adaptation period, the rabbits entered a formal 28-day experimental period, during which daily feed intake of each rabbit was recorded, and weights were measured every 7 days. Using the data on weight changes and food consumption for each stage, the average daily feed intake (ADFI) and average daily gain (ADG) for that stage were calculated accordingly. Upon completion of the experiment, the rabbits were grouped based on feed intake, selecting the top six rabbits in terms of intake to form the High group and the Low group. Student’s t-test was then conducted to validate the difference in feed intake between the two groups. Blood samples were collected from the marginal vein of the ear into anticoagulant (sodium heparin)-containing tubes, centrifuged at 3,000 rpm for 10 minutes. The supernatant was then transferred to 1.5-mL centrifuge tubes and stored long term at −80°C. Each rabbit was euthanized using air embolism. Samples of the stomach, abdominal fat, cecum, hypothalamus, and cecal contents were collected and stored long term at −80°C.

#### Experiment 2: FMT

##### Antibiotic treatment

According to the previous protocol, each rabbit was subjected to antibiotic treatment (18, 19). In brief, ampicillin (1 g/L; A5354, Sigma-Aldrich, Saint Louis, USA), vancomycin (0.5 g/L; SBR00001, Sigma-Aldrich, Saint Louis, USA), metronidazole (1 g/L; M3761, Sigma-Aldrich, Saint Louis, USA), and neomycin (1 g/L; N6386, Sigma-Aldrich, Saint Louis, USA) were added to the rabbits drinking water for a week to deplete the intestinal microbiota completely.

##### Preparation of fecal suspension and experimental design

As previously described, the preparation of fecal suspension was conducted (20–22). Fresh feces from the high intake group rabbits were immediately collected in sterile self-sealing bags and stored at −80°C. The collected feces from the high intake group were weighed, diluted with sterile saline to a concentration of 1 g/5 mL. The suspension was sequentially filtered through stainless steel sieves of 2, 1, 0.5, and 0.25 mm, followed by centrifugation at 6,000 *g* for 15 minutes, discarding the supernatant. The precipitate was resuspended in sterile saline containing 10% glycerol and stored long term at −80°C. Before FMT, the frozen fecal suspension was thawed in a 37°C water bath, diluted with sterile saline to a concentration of 1 × 10^9^ CFU/mL, and administered to the recipient rabbits via gavage at room temperature.

Twelve 30-day-old rabbits with similar feed intake and body weight (679.04 ± 10.21 g) obtained from Wuhan Wanqian Jiaxing Biotechnology Co. Ltd. (Wuhan, China) were randomly divided into two groups: the FMT-Con group and FMT-High group, with six replicates in each group, consisting of one rabbit per replicate. Each rabbit was individually housed in a single cage with *ad libitum* access to feeds and water. After acclimatization for 1 week, each rabbit underwent a 1-week antibiotic treatment according to the method described above. Following another week of normal feeding, the FMT experiment was conducted. The FMT-High group received 2 mL of fecal suspension (1  ×  10^9^ CFU/mL) via gavage daily, while the FMT-Con group received an equivalent volume of sterile saline. During the formal experimental period, the feed intake of each rabbit was recorded daily, and weighing of each rabbit was conducted every 7 days. At the end of the experiment, euthanasia was performed using air embolism, and serum, hypothalamus, stomach, and intestinal tissues were collected for further analysis.

### Experiment 3: GABA gavage experimental design

Twelve rabbits (30 days old, weight 684.34 ± 8.34 g) provided by Wuhan Wanqian Jiaxing Biotechnology Co. Ltd. (Wuhan, China) were randomly divided into the Con group and GABA group, with six replicates in each group, consisting of one rabbit per replicate. Each rabbit was housed individually in a single cage with *ad libitum* access to feeds and water. After acclimatization for 1 week, the GABA group was administered gastric gavage with GABA dissolved in physiological saline at a dose of 10 mg/kg (A2129, Sigma-Aldrich, Saint Louis, USA), while the Con group received an equivalent volume of saline. The feed intake of each rabbit was recorded daily, and weighing of each rabbit was conducted every 7 days. At the end of the experiment, euthanasia was performed using air embolism, and serum was collected for analysis.

### Real-time quantitative PCR (RT-qPCR)

Total RNA was extracted, and RT-qPCR was performed following the established protocol (23). qPCR was conducted using the Accurate Biology SYBR Green Premix Pro Taq HS qPCR Kit (AG11701, Accurate Biotechnology, Changsha, China) as per the manufacturer’s instructions. *Glyceraldehyde-3-phosphate dehydrogenase* (*GAPDH*) was used as the housekeeping gene, and PCR analysis was performed using the 2^−ΔΔCT^ method. The primers used in this study were synthesized by Accurate Biotechnology (Changsha, China), and the sequences are provided in Table S2.

### Hormone detection in serum

According to the manufacturer’s instructions, enzyme-linked immunosorbent assay (ELISA) kits were used to measure the levels of Peptide YY (PYY; YJ027358, Mlbio, Shanghai, China), Glucagon-like Peptide-1 (GLP-1; YJ333690, Mlbio, Shanghai, China), Ghrelin (YJ366258, Mlbio, Shanghai, China), Leptin (YJ932978, Mlbio, Shanghai, China), and Cholecystokinin (CCK; YJ932978, Mlbio, Shanghai, China) in the serum.

### Western blot

Total protein was extracted from the hypothalamus using RIPA lysis buffer containing 1% protease inhibitor (P6730; Solarbio, Beijing, China) and 1% phosphatase inhibitor (P1260; Solarbio, Beijing, China). The lysate mixture was centrifuged at 12,000 rpm for 15 minutes at 4°C, and the supernatant was collected for protein concentration determination using the BCA protein quantification kit (PC0020, Solarbio, Beijing, China). Subsequently, protein separation was performed using 10% SDS-PAGE gel electrophoresis, and the proteins were transferred to a 0.45-µm PVDF membrane (Millipore, MA, USA). The membrane was blocked in 5% skim milk for 1 hour, followed by overnight incubation at 4°C with primary antibodies against Neuropeptide Y (NPY) and Pro-opiomelanocortin (POMC). The next day, secondary antibodies were applied. Chemiluminescent detection was carried out using an enhanced chemiluminescence kit (P0018FM; Beyotime, Shanghai, China), and visualization was performed using a gel imaging system (Uvitec, Cambridge, UK). β-Actin or β-tubulin was used as the housekeeping protein in this experiment. ImageJ v1.8.0 software was used for quantifying the intensity of protein bands. Details of the antibodies used are provided in Table S3.

### Immunofluorescence analysis

Following the established protocol (24), the hypothalamic tissue was fixed with 4% paraformaldehyde, dehydrated in ethanol, and embedded in paraffin. The paraffin-embedded blocks were sectioned into 5–6-μm-thick slices, followed by overnight incubation with the respective primary antibodies. The next day, an HRP-conjugated IgG secondary antibody was applied. Subsequently, the slices were stained with 4′,6-diamidino-2-phenylindole (DAPI) solution (C0065; Solarbio, Beijing, China) at room temperature in the dark for 10 minutes. Imaging was performed using the Eclipse TI-SR fluorescence microscope (Nikon, Tokyo, Japan) and DS-U3 imaging system (Nikon, Tokyo, Japan).

### 16S rRNA high-throughput sequencing

Total DNA was extracted from cecal contents using the E.Z.N.A. Soil DNA Extraction Kit (Omega Bio-tek, Norcross, GA, USA), and the DNA concentration and purity were determined using NanoDrop 2000 (Thermo Scientific, USA). PCR amplification of the full-length 16S rRNA gene was performed using primers 27F (5′-AGRGTTYGATYMTGGCTCAG-3′) and 1492R (5′-RGYTACCTTGTTACGACTT-3′) with barcodes (25), with 10 ng of DNA as the template. Purified amplicons were mixed in appropriate proportions based on the sequencing requirements of each sample. Library construction was carried out using the SMRTbell prep kit 3.0, and sequencing was performed on the PacBio Sequel IIe System (Shanghai Meiji Biomedical Technology Co. Ltd.). HiFi reads were generated from the sequencing subreads using the CCS mode of SMRT-Link v11.0 for subsequent data analysis. Data from each sample were distinguished based on barcode sequences, and sequences of 1,000–1,800 bp were retained. The sequences were clustered into operational taxonomic units (OTUs) at 97% similarity using UPARSE 7.1 (26), and chimeras were removed. OTU species taxonomic annotation was performed using the RDP classifier (v2.11) against the Silva 16S rRNA gene database (v138), and community composition of samples was analyzed at different taxonomic levels (27). Alpha diversity, beta diversity, lefse analysis, picrust2 function prediction analysis, and other analyses were conducted on the Majorbio cloud platform (www.majorbio.com). Differential biomarkers between two groups were identified using the Mann-Whitney U test (non-parametric test) with a threshold of *P* < 0.01 and Linear Discriminant Analysis (LDA) score of 3. Species with Spearman correlation |*R*| > 0.6 and *P* < 0.05 at the genus level were selected for correlation network analysis, visualized using Gephi v0.1 software.

### Measurement of glutamate decarboxylase and GABA

The levels of glutamate decarboxylase (GAD) in cecal contents were determined using an ELISA kit (ml092748, Mlbio, Shanghai, China) following the manufacturer’s instructions.

For the measurement of GABA levels, an High Performance Liquid Chromatography (HPLC) method was employed. 0.15 g of cecal contents and feces was weighed and added to 25 mL of 50% alcohol. The mixture was heated at 70°C for 30 minutes, cooled, and then centrifuged at 12,000 rpm, 4°C for 10 minutes to collect the supernatant. A standard solution of GABA was prepared by dissolving 10 mg of GABA (A2129, Sigma-Aldrich, Saint Louis, USA) in 10 mL of deionized water to make a 1-mg/mL solution. This standard solution was further diluted to different concentrations before derivatization. Two hundred microliters of the sample supernatant or GABA standard solution was mixed with 100 µL NaHCO_3_ (0.5 mol/L, pH 9.0), 20 µL 1% FDBN, and 180 µL deionized water. The mixture was incubated in the dark at 60°C for 1 hour and then cooled, and 400 µL of KH_2_PO_4_ (0.01 mol/L, pH 7.0) was added and mixed. The derivatized product was analyzed using HPLC with an Agilent TC-C18 (5 µm, 4.6 × 250 mm) column at a detection wavelength of 360 nm. The mobile phase consisted of Phase A: 5 mmol/L NaAc (sodium acetate) (pH 5.7, adjusted with 2% HAc), 50 mL of tetrahydrofuran (chromatographically pure) was added and diluted to 1 L with deionized water, and Phase B: methanol (chromatographically pure). The elution gradient program is detailed in Table S4. The GABA standard solution was used for constructing the standard curve.

### Statistical analysis

The data obtained were analyzed using Student’s t-test or the Mann-Whitney U test, and the results were presented as mean ± SEM. Data with 0.05 < *P* < 0.1 were considered to show a trend toward significance, *P < 0.05 indicated significant difference, and **P < 0.01 indicated highly significant difference.

## RESULTS

### Comparison of growth performance between High and Low groups

**Fig 1:**
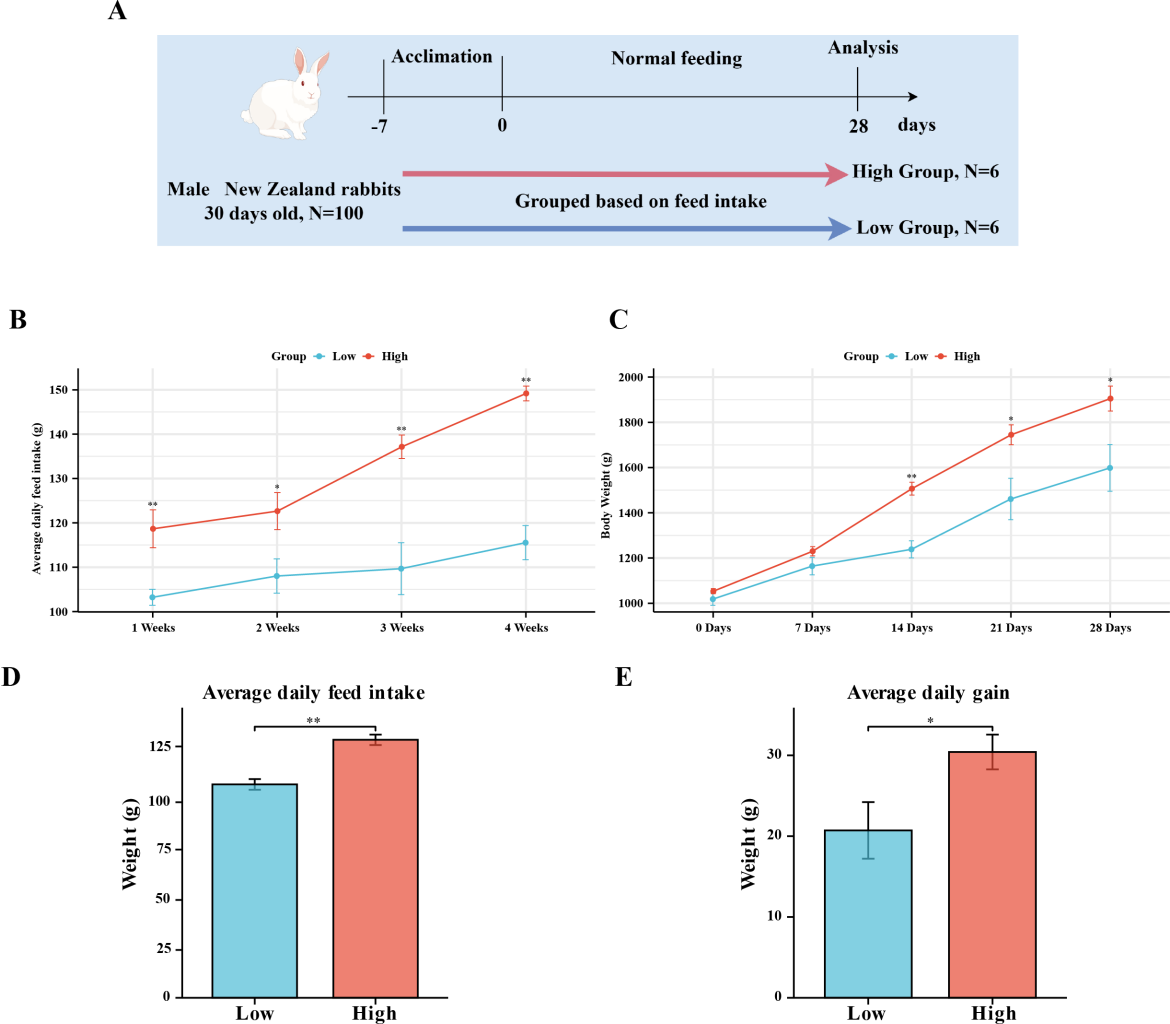
Comparison of body weight and feed intake between the High and Low groups. (**A**) Schematic representation of the feed intake grouping experimental design. (**B**) Weekly changes in the ADFI for both groups. (**C**) Comparison of body weight changes between the two groups. (**D**) ADFI throughout the entire feeding stage for both groups. (**E**) ADG throughout the entire feeding stage for both groups. Data are expressed as the mean ± SEM. *P < 0.05 and **P < 0.01.

In this study, the feed intake during the formal feeding stage of rabbits was used as the criterion for grouping. The feed intake of all 100 rabbits was calculated, and the top six rabbits were selected to form high and low feed intake groups (Fig. 1A). The feed intake and body weight changes of these two groups of rabbits were then analyzed and compared. As shown in Fig. 1B and D, the High group exhibited significantly or highly significantly higher ADFI compared with the Low group on a weekly basis and throughout the feeding stage. When considering body weight changes, with no initial difference in body weight, the two groups showed significant differences in body weight from day 14 to day 28 (Fig. 1C). Furthermore, the High group demonstrated significantly higher ADG throughout the feeding stage compared with the Low group (Fig. 1E). These results indicate a highly significant difference in feed intake between the High and Low groups, confirming the successful construction of the grouping model.

### Comparison of appetite hormone levels between High and Low groups

**Fig 2:**
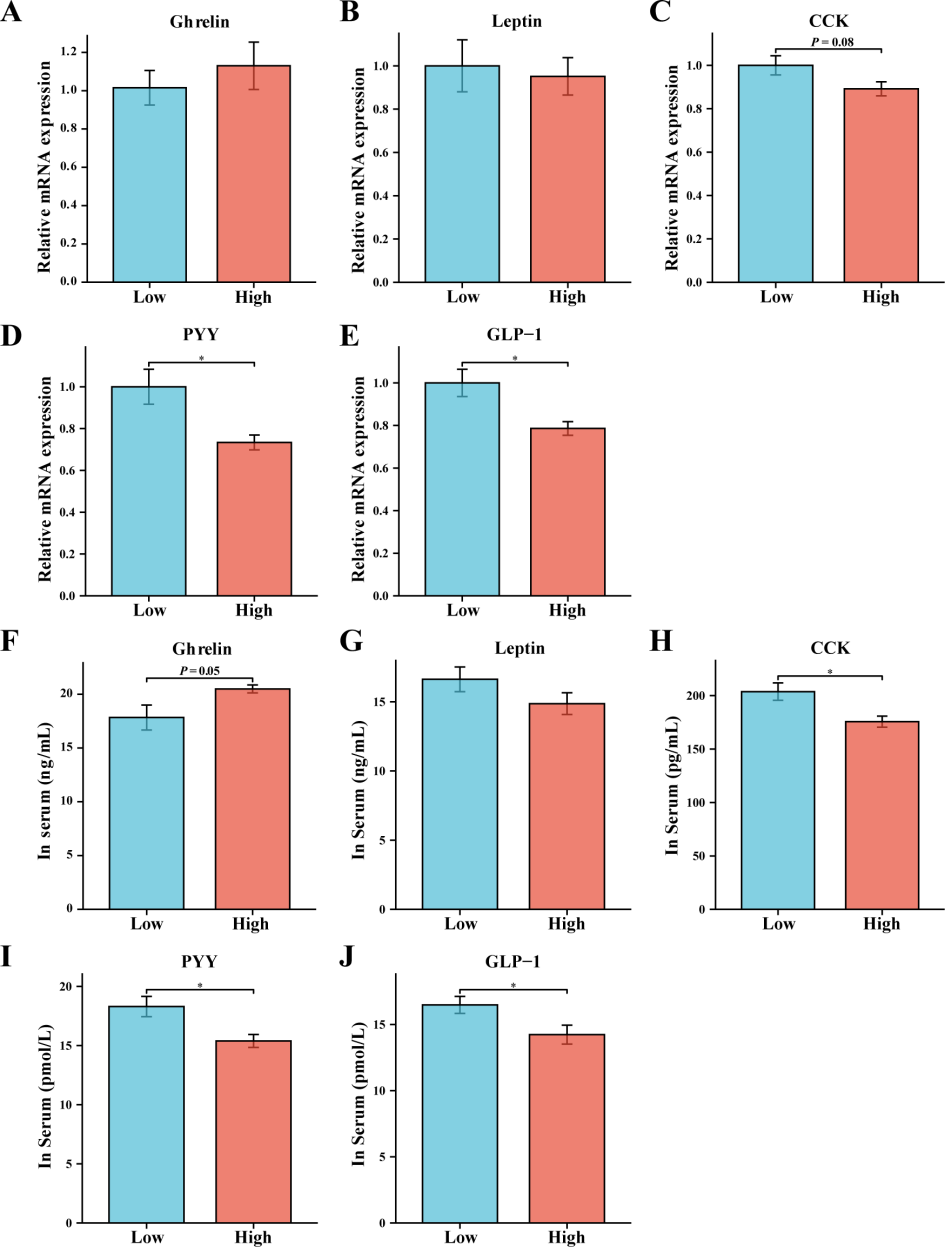
Comparison of appetite hormone levels between High and Low groups. (**A–E**) RT-qPCR measurement of the relative mRNA expression of *Ghrelin*, *Leptin*, *CCK*, *PYY*, and *GLP-1. GAPDH* was used as the housekeeping gene. (**F–J**) Contents of Ghrelin, Leptin, CCK, PYY, and GLP-1 in the serum. Data are expressed as the mean ± SEM. *P < 0.05.

The gastrointestinal tract, which serves as the primary site for digestion and food storage, is capable of sensing changes in the body’s nutritional status and releasing appetite-related hormones into the circulation to regulate feeding behavior (28). In terms of orexigenic hormones, although the relative mRNA expression of *Ghrelin* in the High group was similar to that in the Low group, there was an increasing trend in its serum levels (Fig. 2A and F). On the other hand, regarding anorexigenic hormones, compared with the High group, the Low group exhibited significant increases or increasing trends in the relative mRNA expression and serum levels of GLP-1, PYY, and CCK (Fig. 2C through E, H through J). It is worth noting that there were no significant differences in the relative mRNA expression and serum levels of Leptin between the two groups (Fig. 2B and G), which may suggest that the size of adipose tissue is similar in both the High and Low groups of rabbits.

### Comparison of appetite neuropeptide levels between High and Low groups

**Fig 3:**
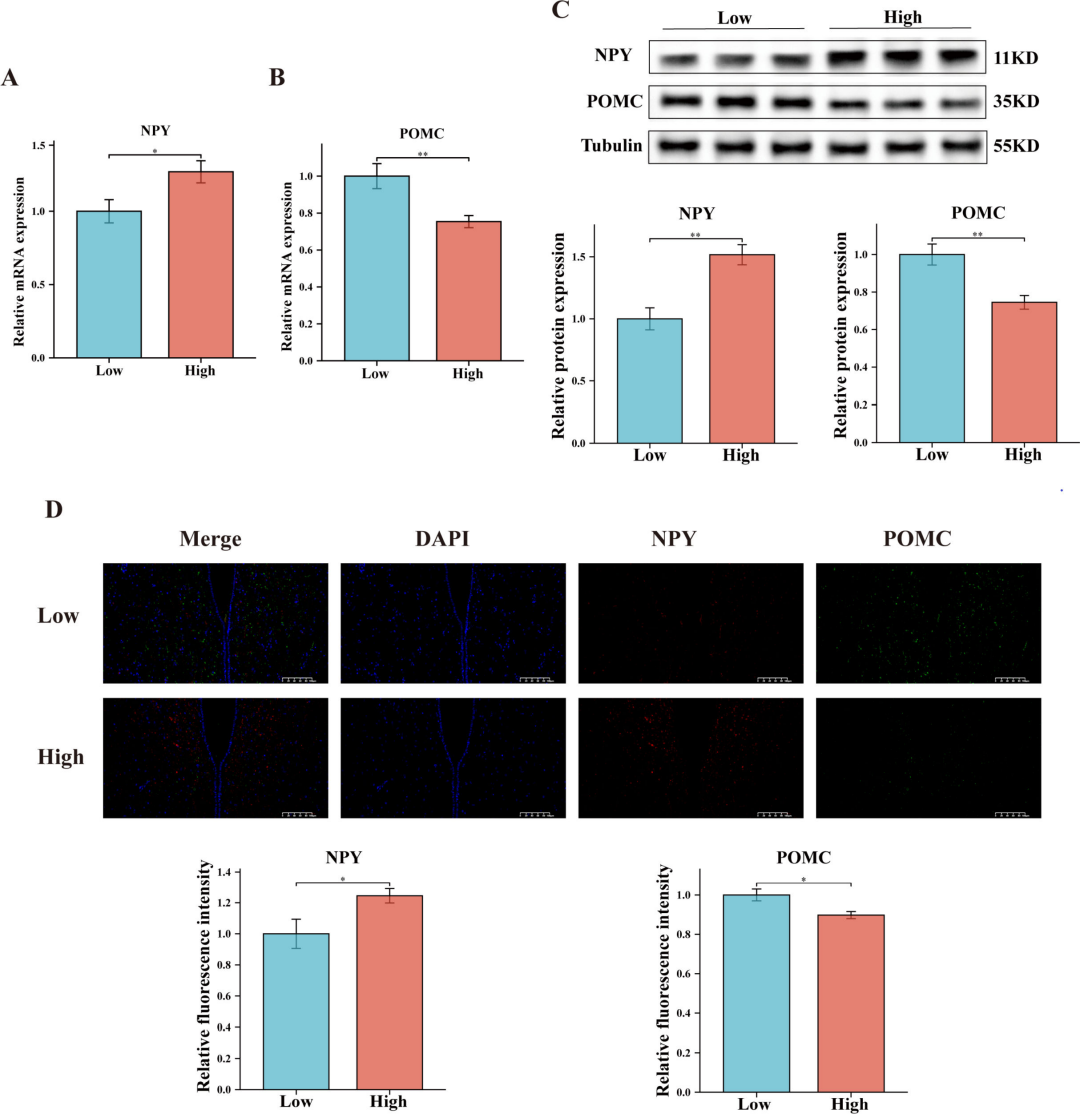
Comparison of appetite neuropeptide expression levels between High and Low groups. (**A and B**) RT-qPCR measurement of the relative mRNA expression of *NPY* and *POMC. GAPDH* was used as the housekeeping gene. (**C**) Western blot technique for determining the relative expression of NPY and POMC proteins in the hypothalamus. Tubulin was used as the housekeeping protein. (**D**) Immunofluorescence staining images of NPY and POMC in the hypothalamus. Data are expressed as the mean ± SEM. *P < 0.05 and **P < 0.01.

The central nervous system serves as the core node for regulating feeding behavior in the organism. Specifically, the NPY and POMC neurons located in the arcuate nucleus (ARC) of the hypothalamus are widely recognized within the field as the “gold standard” for measuring the organism’s appetite (29). RT-qPCR and western blot techniques were employed to detect the expression of NPY and POMC. It was observed that the Low group exhibited lower NPY expression levels and higher POMC expression levels (Fig. 3A through C). Additionally, the results of immunofluorescence staining further confirmed this observation, with significantly higher NPY protein levels and significantly lower POMC protein levels in the High group compared with the Low group (Fig. 3D).

### GABA-producing bacteria were enriched in the high group

**Fig 4:**
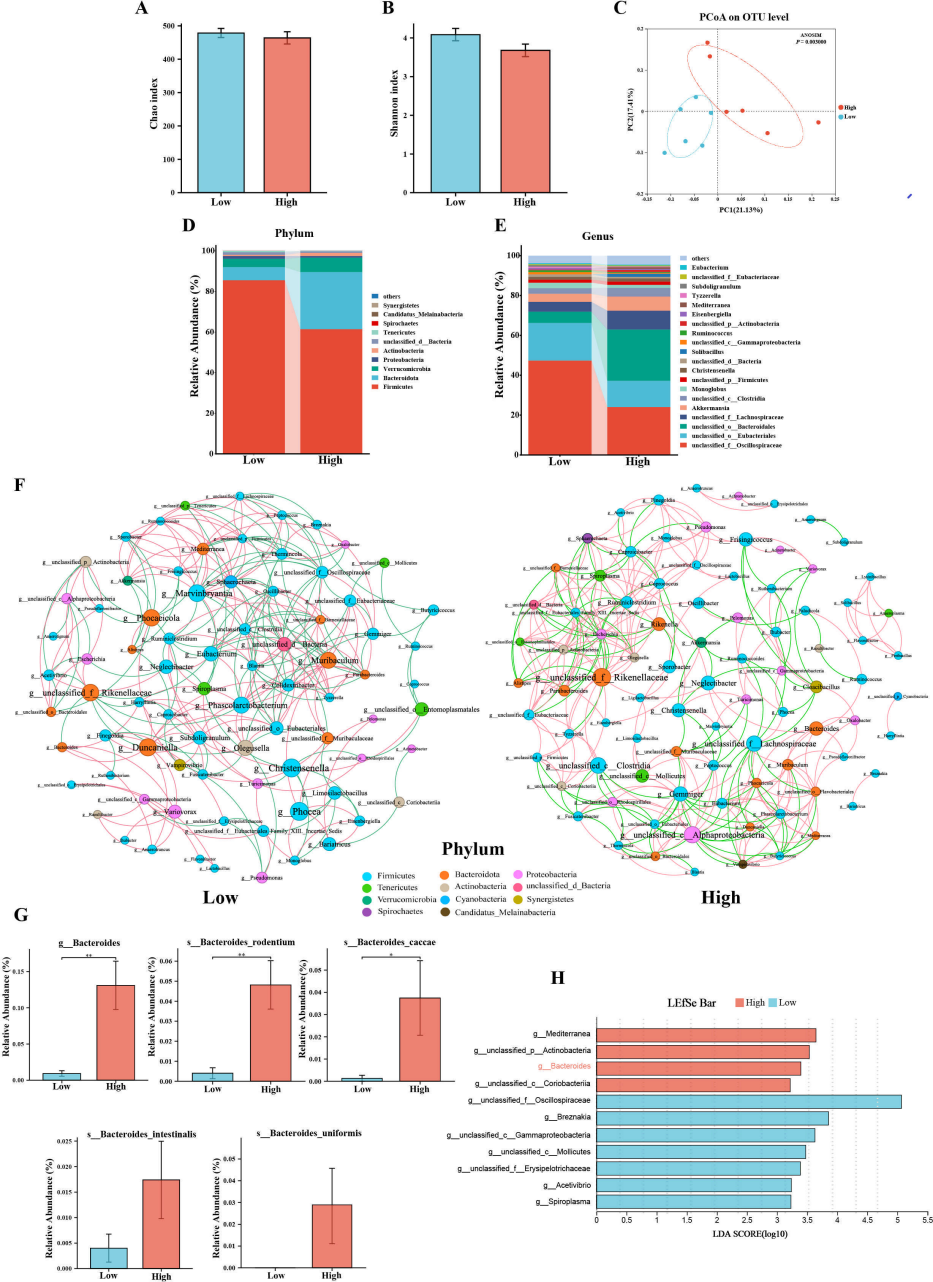
The differences in gut microbiota composition between the High and Low groups. (**A and B**) Differences in species diversity between the two groups assessed using Chao and Shannon index. (**C**) PCoA plot of unweighted UniFrac distances based on relative abundance of OTUs. (**D and E**) Relative abundance of gut microbiota at the phylum level (top 10) and genus level (top 20) for the two groups. (**F**) Spearman’s correlation analysis of the co-occurrence network of gut microbiota based on genus level (with ∣*R*∣ > 0.6 and *P* < 0.05 as thresholds, red for positive correlation and green for negative correlation). (**G**) Relative abundance of *Bacteroides* at genus level and species level. (**H**) LDA effect sizes showing highly significant differences between the two groups of gut microbiota at the genus level. Data are expressed as the mean ± SEM. *P < 0.05 and **P < 0.01.

In order to assess the gut microbiota composition in the two groups, this study conducted 16S rRNA gene sequencing on the cecal contents of rabbits. Fig. S1A and B depict the Rarefaction curves and Shannon curves generated from the gene sequencing data, showing a plateau, indicating that the sequencing data volume was sufficient to cover the majority of bacteria. As shown in Fig. 4A and B, α-diversity indices including Chao and Shannon did not exhibit differences between the two groups. Subsequent β-diversity analysis of the gut microbiota based on unweighted UniFrac Principal Co-ordinates Analysis (PCoA) revealed a significant difference in microbiota composition between the High group and Low group (Fig. 4C). At the phylum level, *p__Firmicutes*, *p__Bacteroidetes*, and *p__Verrucomicrobia* were identified as the main dominant phyla in both groups (Fig. 4D). At the genus level, the main dominant genera in both groups included *g__unclassified_f__Oscillospiraceae*, *g__unclassified_o__Eubacteriales*, *g__unclassified_o__Bacteroidales*, *g__unclassified_f__Lachnospiraceae*, and *g__Akkermansia* (Fig. 4E).

Using ∣*R*∣ > 0.6 and *P* < 0.05 as thresholds, a co-occurrence network analysis of the relative abundance of genus was conducted for the two groups (Fig. 4F). The Low group contained 80 nodes and 218 edges, with modularization and average clustering coefficients of 0.522 and 0.506, respectively. Meanwhile, the High group had 88 nodes and 318 edges, with modularization and average clustering coefficients of 0.587 and 0.592 (Table 1). These results suggest that the gut microbiota in the High group exhibits a more complex microbial interaction pattern. Betweenness centrality is an important measure to assess the significance of nodes in a network graph, with nodes having higher betweenness centrality playing a more critical role in maintaining the overall network structure (30). Based on betweenness centrality, the top 10 genus were selected for each group. The top 10 genus with the betweenness centrality in the Low group were *g__Phocea*, *g__Christensenella*, *g__Duncaniella*, *g__Marvinbryantia*, *g__unclassified_f__Rikenellaceae*, *g__Phocaeicola*, *g__Phascolarctobacterium*, *g__Muribaculum*, *g__Olegusella*, and *g__Eubacterium*, while *g__unclassified__f__ Rikenellaceae*, *g__unclassified__c__Alphaproteobacteria*, *g__unclassified__c__Clostridia*, *g__unclassified__f__Lachnospiraceae*, *g__Gemmiger, g__Neglectibacter*, *g__Bacteroides*, *g__Frisingicoccus*, *g__Christensenella*, and *g__Rikenella*, on the other hand, were the top 10 genera in terms of betweenness centrality in the High group (Table S5). Subsequent differential analysis of the selected genus revealed that only *g__Bacteroides* showed differences between the two groups, with *s_Bacteroides_rodentium* and *s_Bacteroides_caccae* being the different strains at the species level within the *g__Bacteroides* (Fig. 4G; Fig. S1C). Additionally, the gut microbiota’s genus-level LDA discriminant analysis yielded similar results, showing significant enrichment of *g__Bacteroides* in the High group (Fig.4H). In contrast, the genera significantly enriched in the Low group were primarily *g__Breznakia*, *g__Acetivibrio*, and *g__Spiroplasma* (Fig. 4H). Combining betweenness centrality and LDA discriminant analysis results suggests that *g__Bacteroides* may potentially mediate elevated appetite in the host.

**TABLE 1:** Summary of gut microbial network metrics

Item	Group	
	Low	High
Number of nodes	80	88
Number of edges	218	318
Modularity	0.522	0.587
Average clustering coefficient	0.506	0.592

### The high group exhibit higher levels of GABA

**Fig 5:**
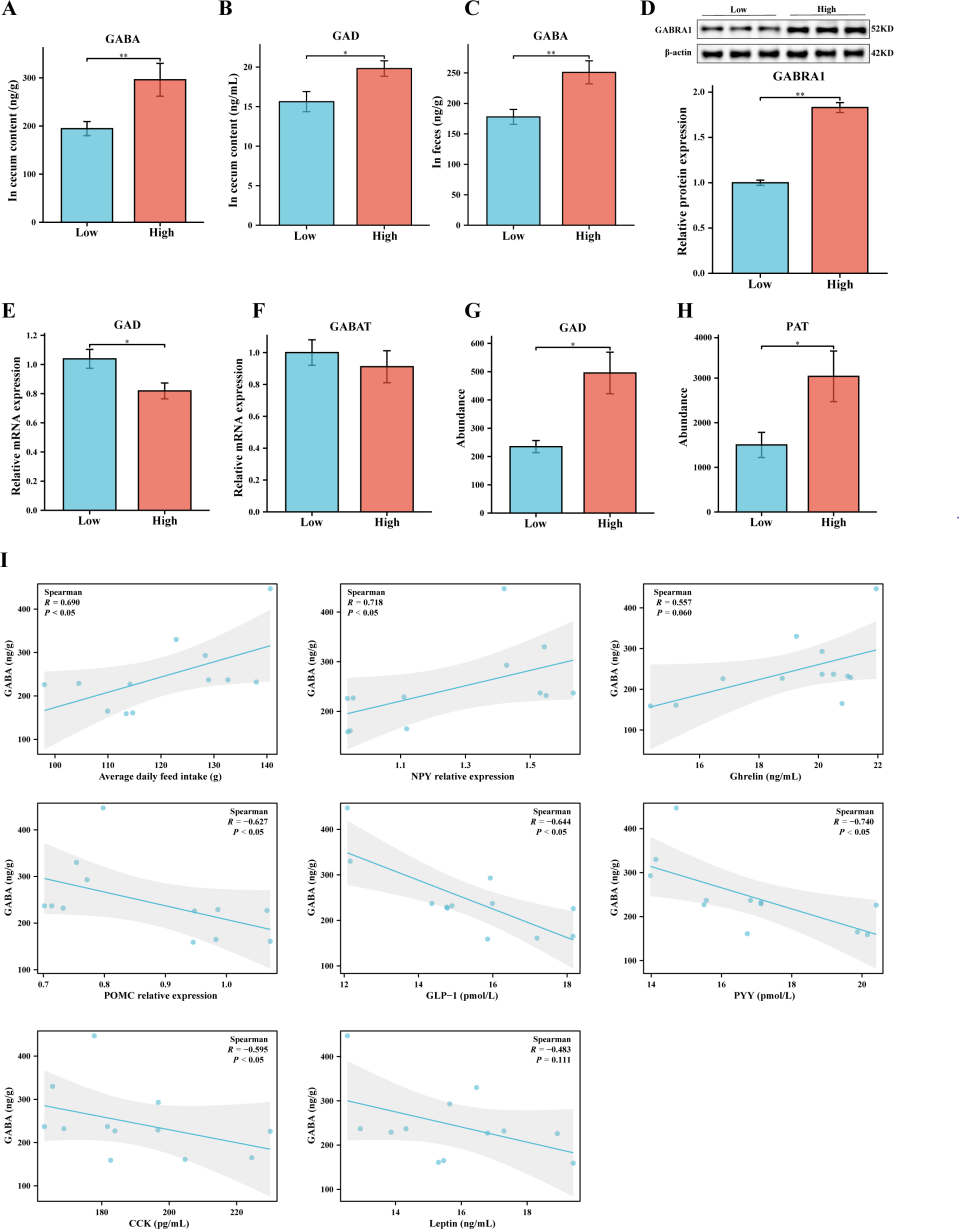
Analysis of gut microbiota-derived GABA of the High and Low groups. (**A and B**) Levels of GAD and GABA in cecal contents. (**C**) Levels of GABA in feces. (**D**) Relative expression of GABRA1 protein in cecal tissue. β-Actin as housekeeping protein. (**E and F**) RT-qPCR measurement of the relative mRNA expression of *GAD* and *GABAT. GAPDH* was used as the housekeeping gene. (**G and H**) Comparison of gut microbiome abundance producing GAD and PAT based on PICRUSt2 functional predictions. (**I**) Spearman correlation analysis between GABA and the ADFI, appetite hormones, and appetite neuropeptides. Data are expressed as the mean ± SEM. *P < 0.05 and **P < 0.01.

There exists a complex and subtle interplay between the gut microbiota and the host, where the metabolic products derived from the microbiota play a crucial role. It has been reported that *g__Bacteroides* can produce GABA to mediate the regulation of bodily functions (31). Therefore, we measured the levels of GABA in two groups, which may be influenced by the release of substances from the gut microbiota. As shown in Fig. 5A and B, the levels of GAD and GABA in the cecal contents of the Low group are significantly or extremely significantly lower than those in the High group. Additionally, the expression levels of the GABRA1 protein exhibit a similarly significant difference trend (Fig. 5D). In order to further confirm that the increase in GABA levels is caused by gut microbiota, we measured the genes of key enzymes [*GAD* and *GABA transaminase* (*GABAT*)] in eukaryotic GABA metabolism in gut tissue, as well as the key enzymes [*GAD* and *putrescine aminotransferase* (*PAT*)] for GABA synthesis in two prokaryotes based on functional predictions (32). It was found that the expression of the *GAD* gene in the Low group was significantly higher than that in the High group, while *GABAT* did not show any differences (Fig. 5E and F). Subsequent functional prediction analyses indicated that the abundance of gut microbiota expressing GAD and PAT enzymes in the High group was significantly higher than that in the Low group (Fig. 5G and H). Therefore, combining these results with the GABA levels in cecal contents and feces (Fig. 5A and C) further confirms that the increased GABA originates from gut microbiota. Subsequently, we employed spearman correlation analysis to explore the relationships between GABA (in cecum contents) and the ADFI, appetite hormones, and hypothalamic appetite neuropeptides. The results indicate a significant or highly significant positive correlation between GABA levels and the relative abundance of NPY levels and ADFI, while showing a significant or highly significant negative correlation with PYY, CLP-1, CCK, and POMC levels (Fig. 5I).

### Effect of FMT-High treatment on rabbit growth performance

In order to further verify that the gut microbiota is a factor leading to differences in host feed intake, we conducted FMT experiments. The experimental design is shown in Fig. S2A, where the gut microbiota from the High group was transplanted into antibiotic-treated rabbits. The results showed that although there was no significant difference in the ADFI between the two groups in the first 2 weeks, the FMT-High group exhibited significantly higher ADFI in the third and fourth weeks compared with the FMT-Con group. Additionally, both groups showed a similar significant difference trend in the ADFI throughout the feeding period (Fig. S2B and D). In terms of weight changes, there were significant differences between the FMT-High group and the FMT-Con group from days 21 to 28, but there was no difference in the ADG between the two groups throughout the feeding period (Fig. S2C and E).

### Effect of FMT-High treatment on the levels of appetite hormones

Compared with the FMT-Con group, the FMT-High group showed a significant decrease in the relative mRNA expression and serum content of CCK, GLP-1, and PYY, while the levels of Ghrelin and Leptin did not exhibit any significant changes (Fig. S3A through J). In summary, FMT-High treatment suppressed the secretion of anorectic hormones. These results further support the notion of a potential connection between the gut microbiota and host appetite regulatory factors.

### Effect of FMT-High treatment on the expression of appetite neuropeptides

Subsequently, we quantified the expression of appetite neuropeptides in the hypothalamus of rabbits after FMT treatment using RT-qPCR, western blot, and immunofluorescence techniques. As shown in Fig. S4A through D, FMT-High treatment significantly upregulated the expression of NPY and downregulated the expression of POMC. These results suggest that FMT-High treatment can promote host appetite by upregulating the expression of orexigenic neuropeptides.

### Effect of GABA treatment on feed intake and anorectic hormones

**Fig 6:**
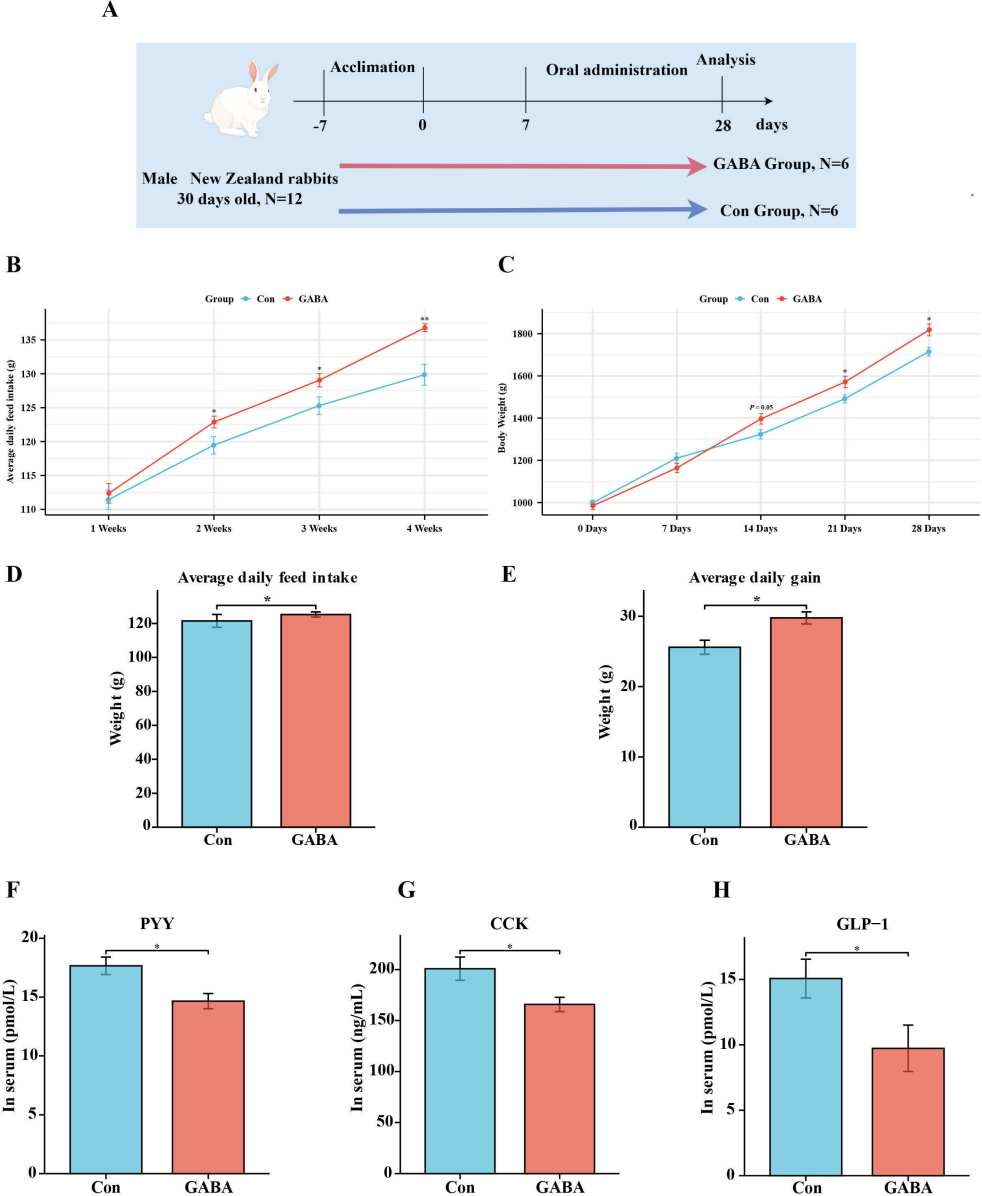
GABA treatment increases feed intake and suppresses the secretion of anorectic hormones. (**A**) Schematic diagram of the GABA gastric gavage experiment. (**B**) Weekly ADFI changes in the GABA group and Control group. (**C**) Weight changes in the GABA group and Control group. (**D**) ADFI throughout the feeding phase in the GABA group and Control group. (**E**) ADG throughout the feeding phase in the GABA group and Control group. (**F–H**) Levels of PYY, CCK, and GLP-1 in the serum of the GABA group and Control group. Data are expressed as the mean ± SEM. *P < 0.05 and **P < 0.01.

To further validate whether GABA can promote feeding behavior, we randomly divided 12 rabbits into the Control group and GABA group. The GABA group received gastric gavage with a dose of 10 mg/kg (dissolved in saline), while the Control group received an equivalent volume of saline solution (Fig. 6A). The results showed that compared with those in the Control group, rabbits in the GABA group had higher feed intake and exhibited a more rapid weight gain (Fig. 6B through E). In terms of appetite hormones, the levels of CCK, GLP-1, and PYY in the GABA group were significantly lower than those in the Control group (Fig. 6F through H). These findings once again support the notion that GABA is negatively correlated with the secretion of anorectic hormones.

## DISCUSSION

As a global issue, loss of appetite has attracted the attention of many researchers. It has been reported that malnutrition resulting from loss of appetite affects approximately 10%–30% of elderly people in Europe and is associated with increased mortality rates (33). Additionally, numerous studies have indicated a close relationship between loss of appetite and the occurrence and development of diseases such as depression, inflammation, and cancer (34–36). Therefore, researchers urgently need to find a new approach to treat loss of appetite. In recent years, with the development of sequencing techniques, increasing evidence suggests that the gut microbiota plays a crucial role in bidirectional communication between the gut and the brain, especially in regulating the host’s appetite (16, 37, 38). Targeting the gut microbiota may be a new strategy for treating loss of appetite. This study utilized 16S rRNA gene sequencing to identify gut microbiota that may influence feeding behavior and further elucidated the underlying molecular mechanisms through fecal transplants and *in vivo* gastric gavage experiments.

This study categorized rabbits into the high feed intake group and low feed intake group based on their feed intake and conducted Student’s t-test on feed intake to validate the successful construction of the grouping model. Compared with the Low group, the High group exhibited a more significant increase in body weight, possibly due to their higher feed intake. Previous studies have indicated that hormones secreted by the gastrointestinal tract play a crucial role as important appetite-regulating factors in the organism’s feeding regulation (39, 40). Zhao et al. demonstrated that supplementing essential oils in feed could increase piglet feed intake by raising serum Ghrelin and Orexin levels (41). Additionally, PYY, GLP-1, and CCK play essential roles in the sensation of satiety and termination of feeding (42). In this study, the High group exhibited higher levels of Ghrelin and lower levels of CCK, GLP-1, and PYY in the serum compared with the Low group. The ARC, as one of the primary targets of these hormones, is located in the basal hypothalamus, with a weak blood-brain barrier and rich blood supply. Therefore, hormones in the peripheral circulation can directly communicate with NPY and POMC appetite-regulating neurons here (43). It is widely believed in the industry that upregulation of NPY neuron activity promotes feeding, while melanocortin signaling expressed by POMC neurons shows the opposite effect (44), consistent with our study results. These data indicate that differences in appetite hormone and neuropeptide expression lead to variations in feed intake between the High and Low groups.

The “microbiota-gut-brain” axis plays an important role in the host’s appetite regulation. To explore the composition of gut microbiota in the High and Low groups, we conducted 16S rRNA sequencing. The PCoA results indicate significant differences in the composition of gut microbiota between the High and Low groups. At the phylum level, the main characteristic of the High group is an increase in relative abundance of *p__Bacteroidetes* and a decrease in *p__Firmicutes*. This phenomenon was also observed in Zhang et al.’s study, where mice with high feed intake exhibited higher relative abundance of *p__Bacteroidetes* and lower relative abundance of *p__Firmicutes* (45). At the genus level, the results from Lesfe show that *g__Breznakia*, *g__Spiroplasma*, and *g__Acetivibrio* are enriched in the Low group. The *g__Breznakia*, as a relatively new bacterial genus, belongs to the *f__ Erysipelothrix*. In cases where the host’s immune system is compromised, certain strains of *f__ Erysipelothrix* can induce inflammation (46), promoting the secretion of inflammatory factors such as TNF-α, which have been proven to have appetite-suppressing effects (47). Similarly, some strains of *g__Spiroplasma* have been found to be pathogenic, especially in insects and other invertebrates, where they can cause disease by infecting the body fluids and tissues (48–50), thereby reducing feed intake. Acetic acid is the primary product of carbohydrate fermentation by *g__Acetivibrio*. Numerous studies have shown that acetic acid can bind to free fatty acid receptor 2 in the intestine, leading to the release of satiety hormones (51). Furthermore, in a scenario where the levels of GLP-1 and PYY in the circulation remain unaffected, intraperitoneal injection of an acetate solution can directly act on anorectic neurons through the blood-brain barrier, inhibiting the host’s feeding behavior (52).

In the co-occurrence network analysis of genus relative abundances, we found that the High group exhibited a more complex microbial interaction pattern. This complexity in microbial interactions may suggest that the host has a higher resistance to foreign infections and a more stable gut microbiota environment, which could be one of the reasons for the lower presence of pathogenic bacteria and higher feed intake in the High group. Subsequently, based on the results of betweenness centrality and Lesfe analysis, we were surprised to discover that *g__Bacteroides* seems to play a crucial role in promoting the host’s feeding behavior. A report evaluating the composition of gut microbiota in healthy elderly individuals with good and poor appetite indicated a significant decrease in the abundance of *g__Bacteroides* in the group with poor appetite (53), which aligns well with our research findings. Considering that *g__Bacteroides* has high betweenness centrality and is significantly enriched in the High group, these results suggest that *g__Bacteroides* may be a key genus in enhancing the host’s appetite.

The gut microbiota can regulate the organism’s energy homeostasis by participating in glutamate metabolism (54). Most *g__Bacteroides* possess the *GAD* gene, which encodes GAD to synthesize GABA (55). Our data indicate that the High group’s cecal contents have higher levels of GAD and GABA, which may be related to the enrichment of *g__Bacteroides* in the High group. Additional studies have indicated that a large number of GABA receptors are distributed on enteroendocrine cells and GABA can influence the physiological functions of the organization by promoting the secretion of signaling molecules by enteroendocrine cells (56). Based on the Spearman correlation analysis and the higher levels of GABA in the High group, we speculate that the increased GABA content in the High group may be the reason for its lower levels of satiety hormones. Wang et al.’s study also confirms our speculation, showing that supplementation of GABA in growing lambs significantly increases the feed intake of livestock and leads to lower levels of CCK in the serum (57). Additionally, numerous studies support the idea that GABA can influence feeding behavior by regulating the expression of appetite-related factors (58, 59). Furthermore, adding GABA to feed can enhance the anti-inflammatory and antioxidant capabilities, thereby improving the growth performance of livestock (60, 61). Taking into account the fact that gut-derived GABA cannot directly affect appetite-regulating neurons through the blood-brain barrier and considering our research findings, we hypothesize that gut microbiota-derived GABA likely increases the host’s appetite by inhibiting the secretion of anorectic hormones.

As a common method in gut microbiota research, FMT is considered by researchers as a means to unveil the causal relationship between gut microbiota and host phenotype changes. In rabbits transplanted with the gut microbiota from the High group, we observed increased feed intake and changes in body weight. Subsequent gavage experiments further validated the negative correlation between GABA and satiety hormones. In conclusion, the results of FMT and GABA gavage experiments once again strongly support the possibility that GABA derived from gut microbiota (especially *g__Bacteroides*) may promote feeding behavior in the host by inhibiting the secretion of anorectic hormones.

### Conclusion

Our data suggest that differences in the composition of the gut microbiota lead to varying feed intake levels. The High group is enriched with bacteria that produce GABA (such as *g__Bacteroides*), while the Low group is characterized by harmful bacteria (such as *g__Breznakia* and *g__Spiroplasma*) and acetate-producing bacteria (*g__Acetivibrio*). Additionally, our study indicates that the potential mechanism behind increased feed intake may be through the inhibition of satiety hormone (GLP-1, PYY, and CCK) secretion by GABA derived from gut microbiota, leading to the upregulation of orexigenic neuron activity. These findings underscore the interactions between gut microbiota and host feeding behavior and provide important insights for the development of future strategies targeting the gut microbiota for the treatment of appetite loss. While our study provides solid evidence supporting the critical role of gut microbiota-derived GABA in promoting feeding behavior, it does have certain limitations. This experiment only used male animals as subjects, and the FMT part did not explore the impact of gut microbiota from the Low group on host feeding changes. Therefore, further research is needed to investigate gender differences and the effects of microbiota from the Low group on host feeding behavior.

## Data Availability

The raw sequence data reported in this paper have been deposited in the Genome Sequence Archive (Genomics, Proteomics & Bioinformatics 2021) in National Genomics Data Center (Nucleic Acids Res 2022), China National Center for Bioinformation / Beijing Institute of Genomics, Chinese Academy of Sciences (GSA: CRA016898) that are publicly accessible at https://ngdc.cncb.ac.cn/gsa.

## SUPPLEMENTAL MATERIAL

Graphical abstract msystems.01015-24-s0001.tiff Graphical abstract.

Supplemental material msystems.01015-24-s0002.docx Fig. S1 to S4 and Tables S1 to S5.

ASM does not own the copyrights to Supplemental Material that may be linked to, or accessed through, an article. The authors have granted ASM a non-exclusive, world-wide license to publish the Supplemental Material files. Please contact the corresponding author directly for reuse.
