# Supplementary material for: Gut microbiota-derived gamma-aminobutyric acid improves host appetite by inhibiting satiety hormone secretion
Source: mSystems. 2024 Sep 24;9(10):e01015-24. doi: 10.1128/msystems.01015-24 (PMC11495008; doi:10.1128/msystems.01015-24)
Supplement: Supplemental material — Fig. S1 to S4 and Tables S1 to S5. [file msystems.01015-24-s0002.docx]

| **Ingredients** | **Content (%)** | **Nutrient levels^2^** | **Content** | |
| --- | --- | --- | --- | --- |
| Corn | 14 | Digestible Energy (MJ/Kg) | | 9.94 |
| Soybean meal | 12 | Dry Matter (%) | | 91.33 |
| Wheat meal | 13 | Crude Protein (%) | | 15.67 |
| Alfalfa meal | 6.5 | Ash (%) | | 7.46 |
| Rice bran | 15 | Ether Extract (%) | | 4.12 |
| Wheat middlings | 8 | Crude Fibers (%) | | 15.04 |
| Soybean husk powder | 15 | Ca (%) | | 0.86 |
| Germ meal | 3.5 | P (%) | | 0.55 |
| Bean straw powder | 9 |  | |  |
| Premix^1^ | 4 |  | |  |
| Total | 100 |  | |  |

**Table S1.** Composition and nutrient levels of basic diet (Air-dry basis)

Note: ^1^ Premix for each kilogram of diet provides: Vitamin D3 1000 IU, Vitamin A 8000 IU, Vitamin E 50mg, Methionine 1.5 g, Lysine 1.5 g, Cu 50mg, Fe 100 mg, Mn 30 mg, Mg 150mg, I 0.1 mg, Se 0.1 mg.

^2^ Nutrient levels were measured.

**Table S2**. Sequences of primers used in real-time quantitative PCR (RT-qPCR)

| **Genes** | **Sequences（5' to 3'）** |
| --- | --- |
| *Ghrelin* (*Ghrl*) | Forward: CGGTAAAGGAAGCAGACAAGG  Reverse: GCGGAAGGTAGAACTAGAGGGA |
| *PYY* | Forward: AGCTGAACCGCTACTACGCC  Reverse: GCCTTCTGACCGAGACCTGA |
| *CCK* | Forward: GCGTGCTGATAGCGGTATTG  Reverse: TCGGGACTCGACATCCGTC |
| *Leptin* | Forward: GCCCTGTCTGTCCTGTGTT  Reverse: ACCGACTGCGTGTGTGAG |
| *GLP-1* (*Gcg*) | Forward: GGATTGTTTGTAATGCTGG  Reverse: TGTCTTCGGTCATCTGGT |
| *NPY* | Forward: GCCCTGTCCCTGCTTGTA  Reverse: TGATGAGGTTGATGTAGTGTCG |
| *POMC* | Forward: CAAGCCGGTGGGCAAGAA  Reverse: TTGACCTCGACGGGGAAGG |
| *GAD* | Forward: CGCTCTCTGTCTGGCTGTACG  Reverse: ACAGTTGTGAGCCTGGTCACTT |
| *GABAT* | Forward: CTTCCGTCTTCATCAGAGGC  Reverse: CAGCTTCCAGCACAGCTACC |
| *GAPDH* | Forward: CACTTCGGCATTGTGGAG  Reverse: GAGGCAGGGATGATGTTCT |

| **Antibody name** | **Company** | **Product category** | **Application** |
| --- | --- | --- | --- |
| NPY | Proteintech Group, Wuhan, China | Cat No: 12833-1-AP | WB, IF |
| POMC | Proteintech Group, Wuhan, China | Cat No: 66358-1-Ig | WB, IF |
| GABRA1 | Proteintech Group, Wuhan, China | Cat No: 12410-1-AP | WB |
| β-actin | Proteintech Group, Wuhan, China | Cat No: 20536-1-AP | WB |
| α-Tubulin | Proteintech Group, Wuhan, China | Cat No: 11224-1-AP | WB |

**Table S3**. List of the primary antibodies used for Western blot (WB) and immunofluorescence (IF) analysis

**Table S4**. Gradient elution protocol

| **Time (min)** | **Mobile phase A%** | **Mobile phase B%** |
| --- | --- | --- |
| 0.01 | 80 | 20 |
| 35 | 40 | 60 |
| 35.01 | 80 | 20 |
| 40 | 80 | 20 |

**Table S5**. Betweenness centrality measured of key bacteria in the gut microbial network

| **Low group** | | **High group** | |
| --- | --- | --- | --- |
| **Genus** | **Betweenness centrality** | **Genus** | **Betweenness centrality** |
| *g__Phocea* | 0.093 | *g__unclassified_f__Rikenellaceae* | 0.090 |
| *g__Christensenella* | 0.093 | *g__unclassified_c__Alphaproteobacteria* | 0.076 |
| *g__Duncaniella* | 0.085 | *g__unclassified_c__Clostridia* | 0.071 |
| *g__Marvinbryantia* | 0.082 | *g__unclassified_f__Lachnospiraceae* | 0.069 |
| *g__unclassified_f__Rikenellaceae* | 0.080 | *g__Gemmiger* | 0.060 |
| *g__Phocaeicola* | 0.079 | *g__Neglectibacter* | 0.057 |
| *g__Phascolarctobacterium* | 0.076 | *g__Bacteroides* | 0.052 |
| *g__Muribaculum* | 0.075 | *g__Frisingicoccus* | 0.052 |
| *g__Olegusella* | 0.069 | *g__Christensenella* | 0.049 |
| *g__Eubacterium* | 0.061 | *g__Rikenella* | 0.047 |


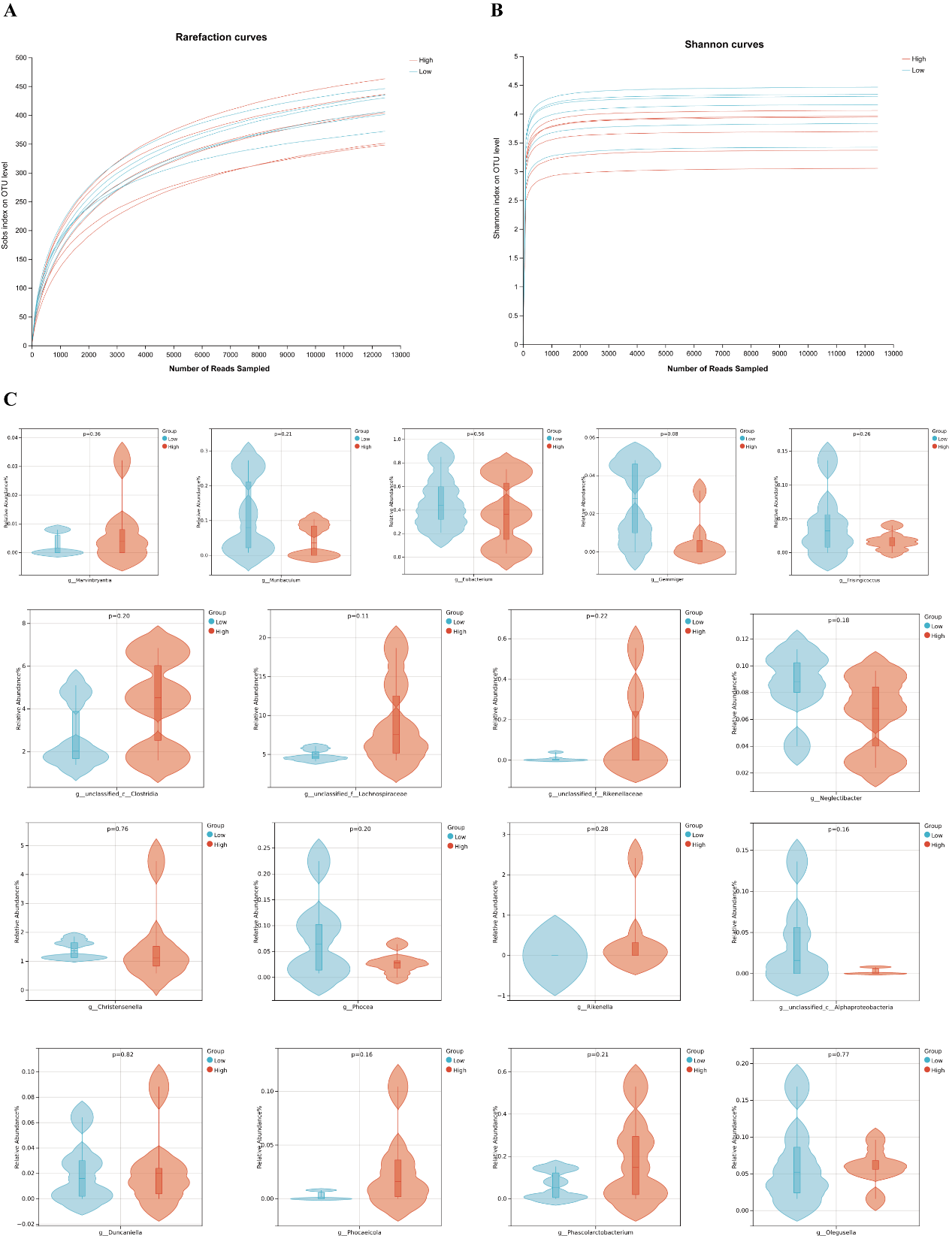


Fig.S1. The relative abundance of top 10 bacteria with betweenness centrality at genus level between High and Low groups. (A)Sob curve. (B)Shannon curve. (C) Comparison of the relative abundance of the Top10 bacteria genus with betweenness centrality in the two groups


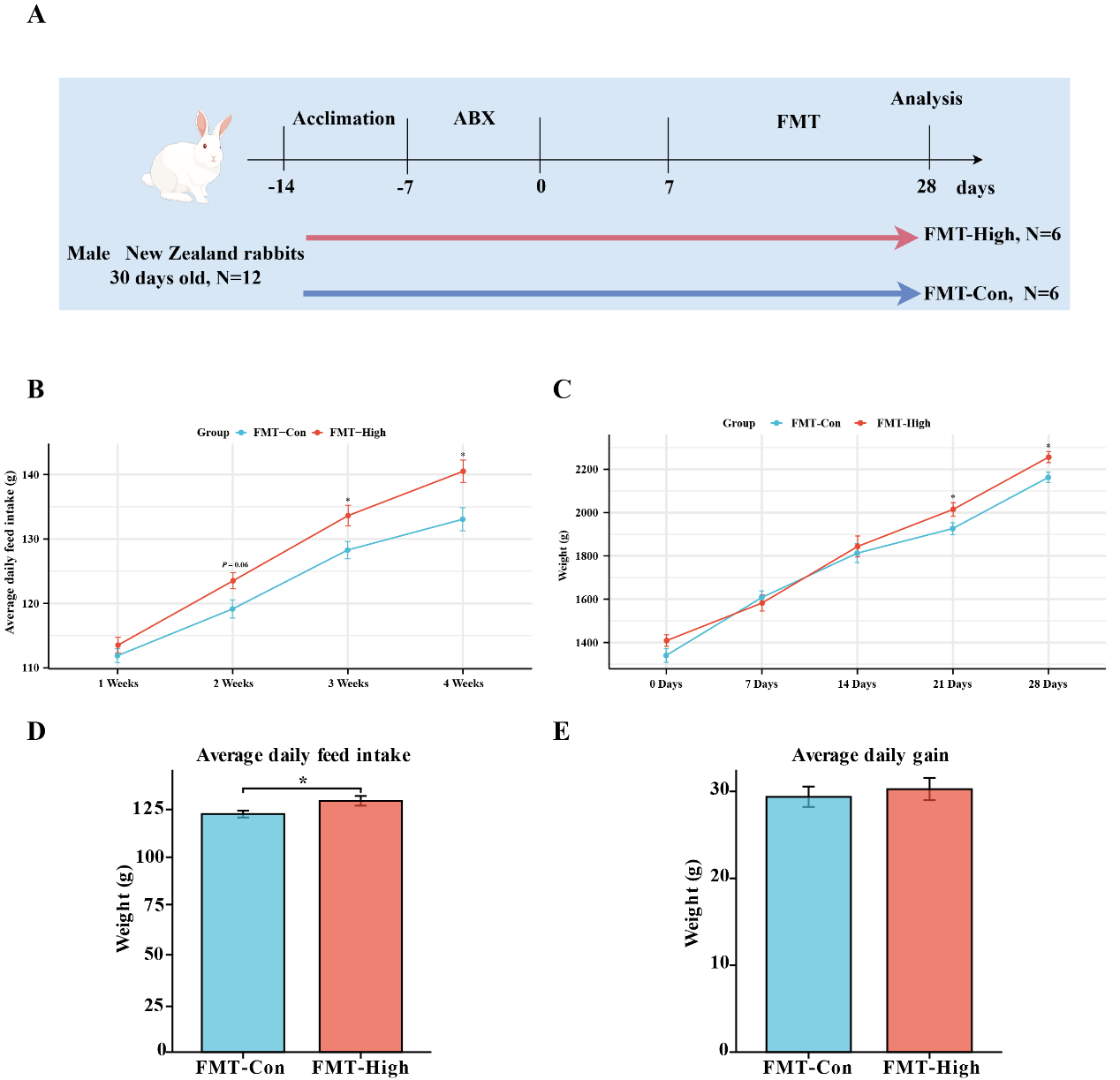


Fig.S2. FMT-High treatment increased host feed intake and weight. (A) Schematic diagram of the FMT experiment design. (B) Weekly changes in ADFI for the FMT-High group and FMT-Con group. (C) Weight changes for the FMT-High group and FMT-Con group. (D) ADFI throughout the feeding period for the FMT-High group and FMT-Con group. (E) ADG throughout the feeding period for the FMT-High group and FMT-Con group. Data are expressed as the Mean ± SEM.**P* < 0.05.


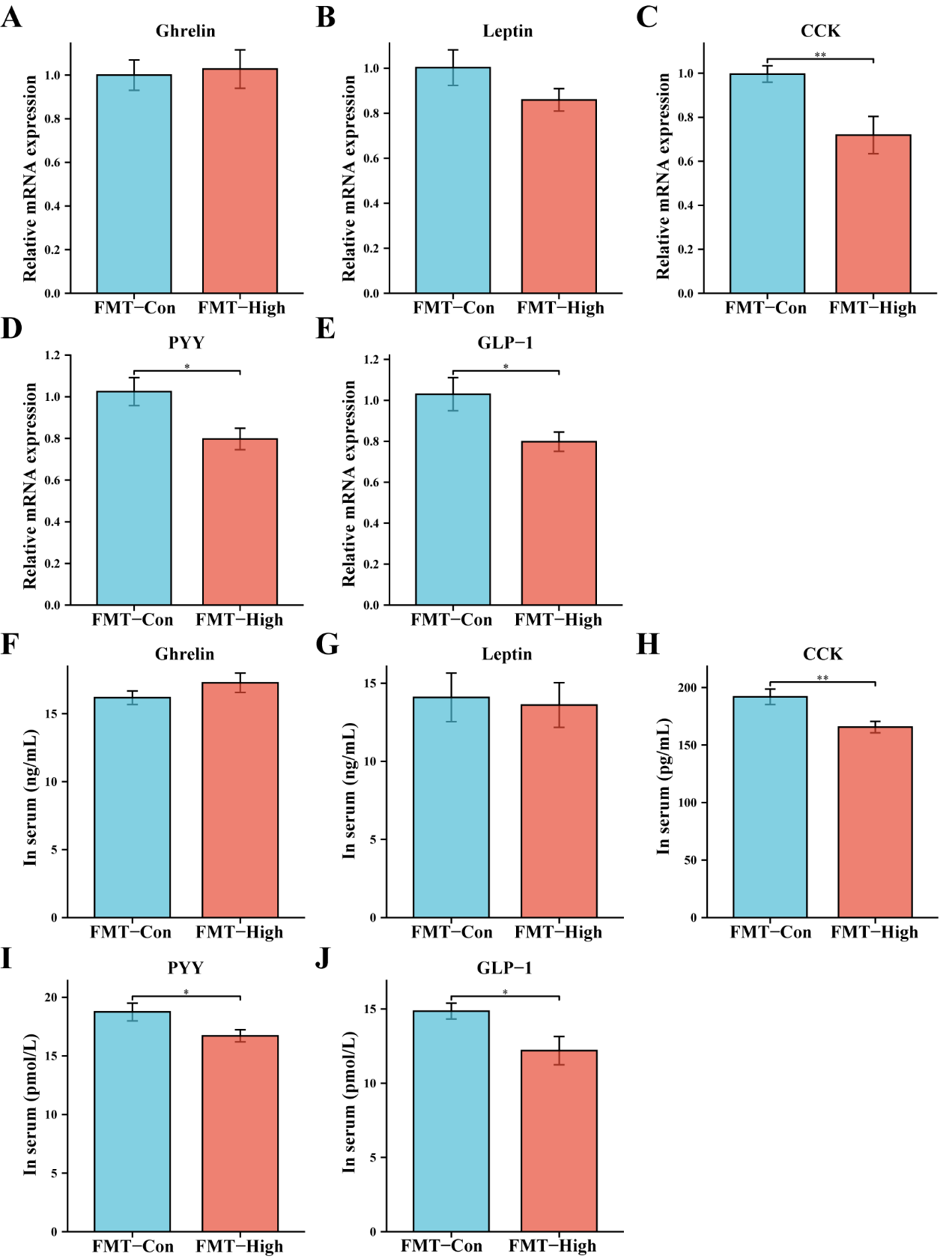


Fig.S3. FMT-High treatment suppressed the secretion of anorectic hormones. (A-E) RT-qPCR measurement of the relative mRNA expression of *Ghrelin*, *Leptin*, *CCK*, *PYY*, and *GLP-1*. *GAPDH* was used as the housekeeping gene. (F-J) Contents of Ghrelin, Leptin, CCK, PYY, and GLP-1 in the serum. Data are expressed as the Mean ± SEM. **P* < 0.05, ***P* < 0.01.


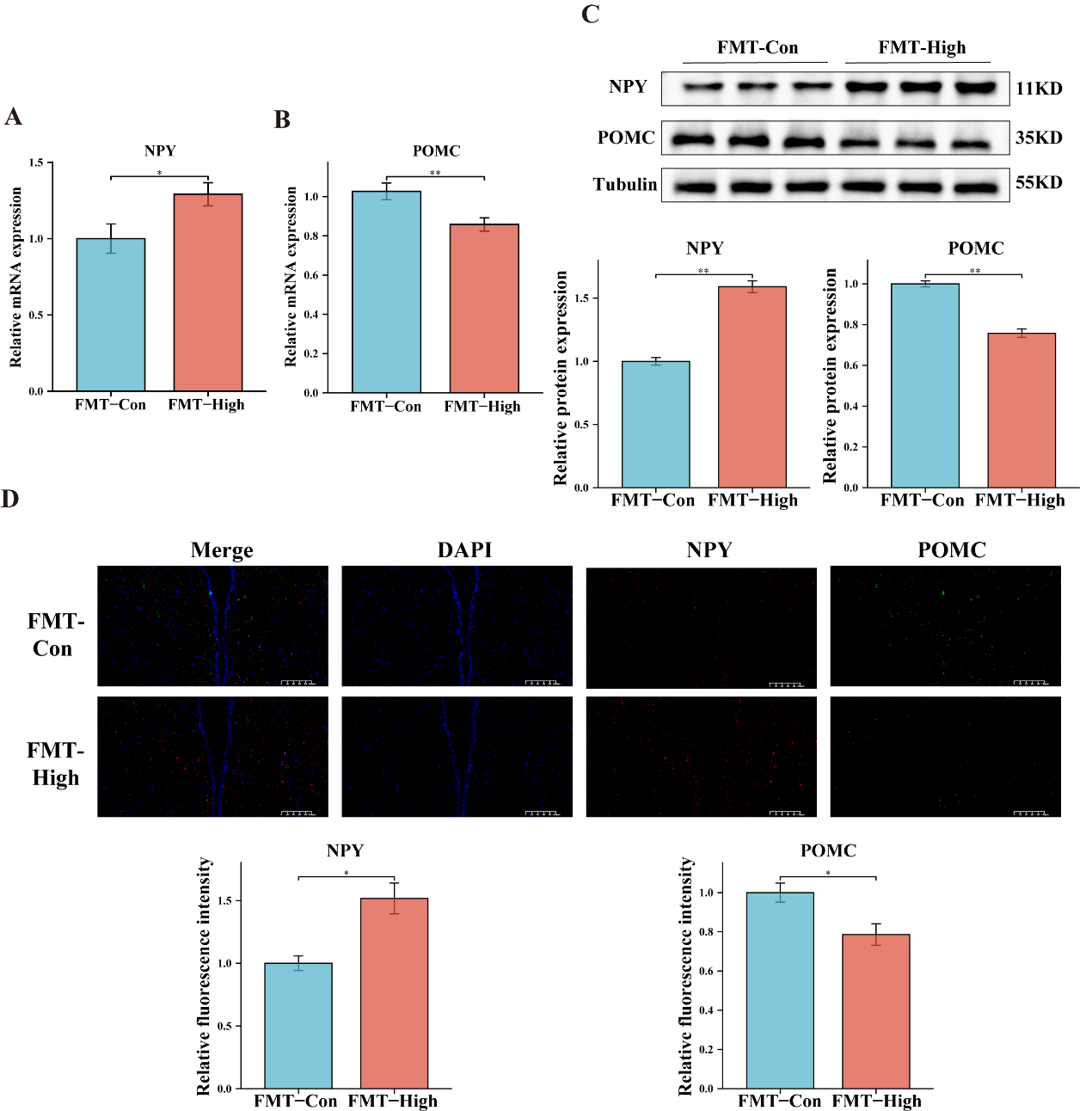


Fig.S4. FMT-High treatment upregulated the expression of orexigenic neuropeptides. (A-B) RT-qPCR measurement of the relative mRNA expression of *NPY* and *POMC*. *GAPDH* was used as the housekeeping gene. (C) Western blot technique for determining the relative expression of NPY and POMC proteins in the hypothalamus. Tubulin was used as the housekeeping protein. (D) Immunofluorescence staining images of NPY and POMC in the hypothalamus. Data are expressed as the Mean ± SEM.**P* < 0.05, ***P* < 0.01.
